# Supplementary material for: Pre- and Post-Zygotic Barriers Contribute to Reproductive Isolation and Correlate with Genetic Distance in Cucumis
Source: Plants (Basel). 2023 Feb 17;12(4):926. doi: 10.3390/plants12040926 (PMC9963866; doi:10.3390/plants12040926)
Supplement: Supplementary file 1 [file plants-12-00926-s001.zip › plants-2082980-supplementary.pdf]

## Supplementary Data

**Supplementary Figure S1.** Evaluation of pre-mating factors in *Cucumis spp*. Box-whisker plots show variability of pollen grain size (A) and stigma (B), style (C) and ovary (D) lengths in the six *Cucumis* species crossed in this work: ANG (*Cucumis anguria*); DIP (*C. dipsaceus*); ZEY (*C. zeyheri*); FIC (*C. ficifolius*); PUS (*C. pustulatus*) and MEL (*C. melo*). Means marked by the same non-capital letter are not statistically different (P>0.05; ANOVA test, see Methods). Feret´s diameter determined by ImageJ software (the longest distance between two points along the boundary of the projected area of pollen grains) was used to estimate pollen grain size. Stigma, style and ovary lengths were estimated by measuring 10 pistils. Pollen grain size was estimated by measuring ~200-300 individual grains.

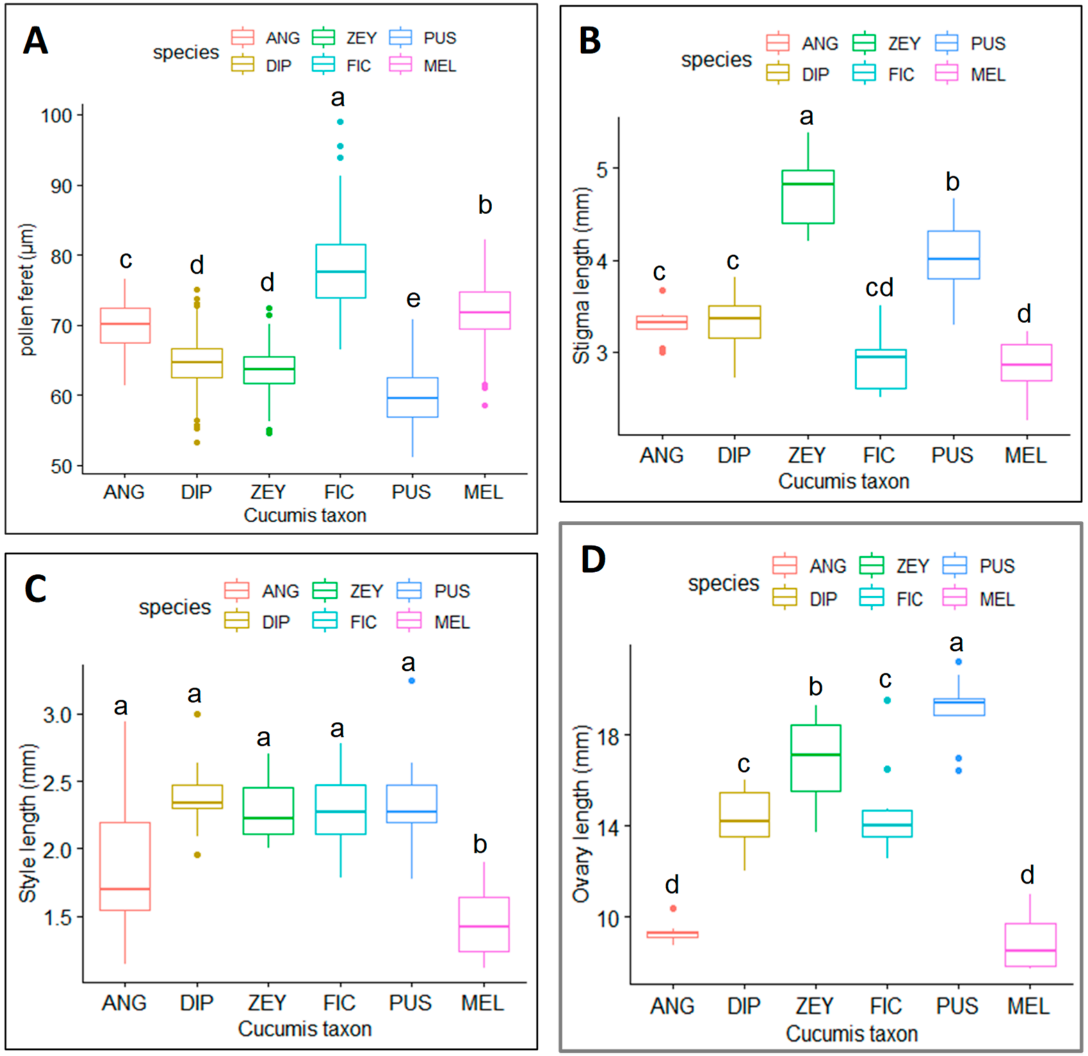

A
species
ANG
DIP
ZEY
FIC
PUS
MEL
pollen feret (µm)
100
90
80
70
60
50
c
d
d
a
e
b
ANG DIP ZEY FIC PUS MEL
Cucumis taxon
B
species
ANG
DIP
ZEY
FIC
PUS
MEL
Stigma length (mm)
5
4
3
c
c
a
cd
b
d
ANG DIP ZEY FIC PUS MEL
Cucumis taxon
C
species
ANG
DIP
ZEY
FIC
PUS
MEL
Style length (mm)
3.0
2.5
2.0
1.5
a
a
a
a
a
b
ANG DIP ZEY FIC PUS MEL
Cucumis taxon
D
species
ANG
DIP
ZEY
FIC
PUS
MEL
Ovary length (mm)
18
14
10
d
c
b
c
a
d
ANG DIP ZEY FIC PUS MEL
Cucumis taxon

**Supplementary Figure S2.** Post-zygotic IRBs in *Cucumis spp*. Histograms show the estimation of four parameters: (A) fruit set (%), (B) fruit weight (g), (C) seed number/fruit and (D) average seed weight (mg) and (E) germination rate (%). Average ± standard error was estimated for fruit weight, seed number/fruit and average seed weight from >10 fruits except for F×P (4) and (D×Z)×Z (5). In addition, only one fruit was obtained from D×A and (D×Z)×D crosses. Means marked by the same non-capital letter are not statistically different (P>0.05; ANOVA test, see Methods). See also Supplementary Table S1 for taxa abbreviations. Ø symbol indicates self-pollination.

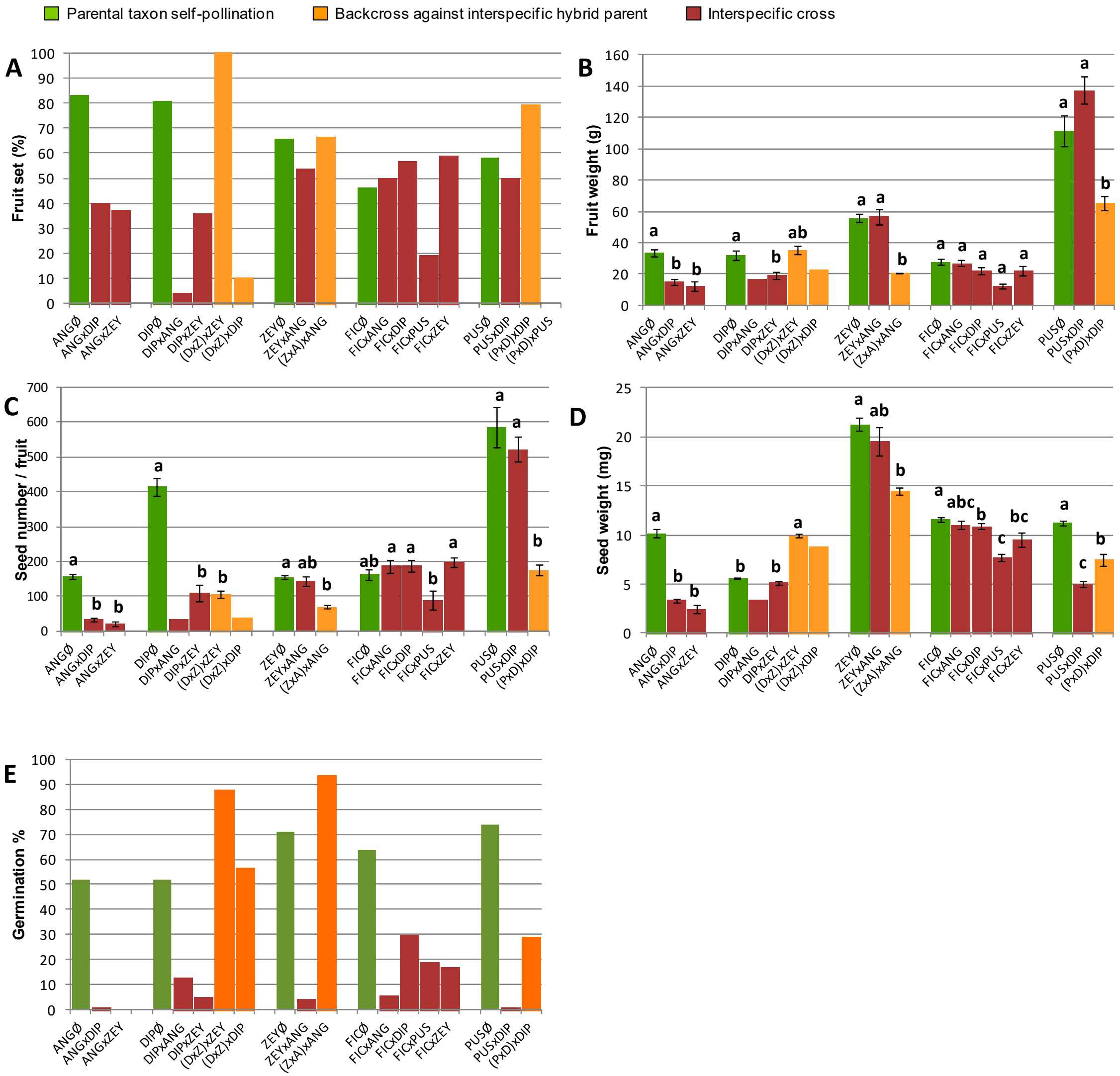

Parental taxon self-pollination
Backcross against interspecific hybrid parent
Interspecific cross
A
Fruit set (%)
B
Fruit weight (g)
C
Seed number / fruit
D
Seed weight (mg)
E
Germination %
ANGØ
ANGxDIP
ANGxZEY
DIPØ
DIPxANG
DIPxZEY
(DxZ)xZEY
(DxZ)xDIP
ZEYØ
ZEYxANG
(ZxA)xANG
FICØ
FICxANG
FICxDIP
FICxPUS
FICxZEY
PUSØ
PUSxDIP
(PxD)xDIP
(PxD)xPUS

**Supplementary Figure S3.** Some of the diagnostic traits (leaf shape, stems and aculei) assessed in the *C. ficifolius* (FIC) and *C. prophetarum* (PRO) accessions used in this work.

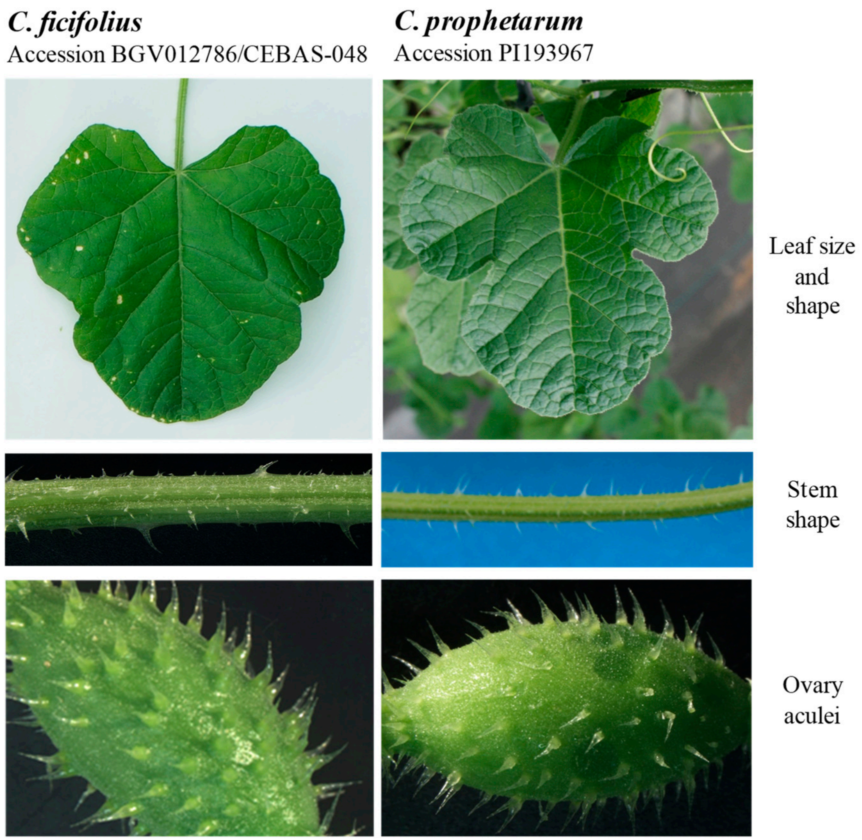
C. ficifolius
Accession BGV012786/CEBAS-048
C. prophetarum
Accession PI193967
Leaf size and shape
Stem shape
Ovary aculei

**Supplementary Figure S4.** Flow cytometry profiles for DIP, FIC and the F×D hybrid. Peaks corresponding to 2C, 3C, 4C, 6C and 8C nuclear DNA content are indicated. All three profiles show peaks of progressively decreasing magnitude. Typically the first one corresponds to nuclei in the G1 phase of mitosis while the second (and eventually the third) corresponds to nuclei in the G2 phase plus those under endomitotic DNA replication.

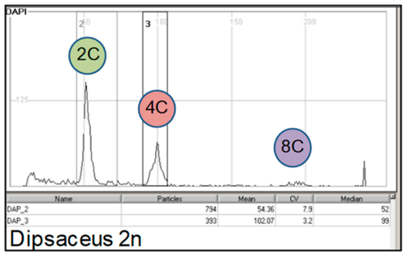


| Name | Particles | Mean | CV | Median |
|---|---|---|---|---|
| DAP_2 | 794 | 54.36 | 7.9 | 52 |
| DAP_3 | 393 | 102.07 | 3.2 | 99 |

Dipsaceus 2n

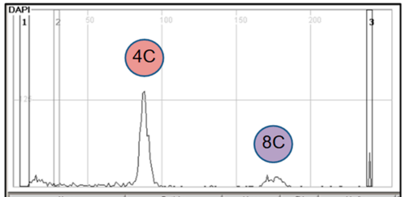


| Name | Particles | Mean | CV | Median |
|---|---|---|---|---|
| DAP_1 | 4 | 10.00 | 8.7 | 10 |
| DAP_2 | 7 | 29.00 | 4.4 | 29 |
| DAP_3 | 49 | 240.00 | 0.0 | 240 |

Ficifolius 4n

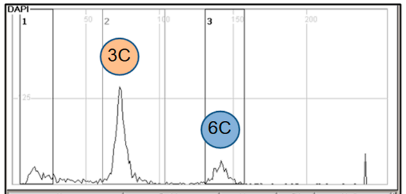


| Name | Particles | Mean | CV | Median |
|---|---|---|---|---|
| DAP_1 | 267 | 17.79 | 27.3 | 17 |
| DAP_2 | 1168 | 75.85 | 7.5 | 74 |
| DAP_3 | 320 | 144.43 | 3.4 | 142 |

Fici x Dip 3n

**Supplementary Table S1.** Plant material used for the GBS analysis. All the scientific names of taxa were assigned according to [46]. Ploidy was determined by flow cytometry (see Methods).

| Species | Accessions | Seed source[a] | Origin | Ploidy | Abbr.[b] |
|---|---|---|---|---|---|
| *C. anguria* L.* | PI282442 *<br>CUC27/1983 | NCRPIS (IRTA)<br>IPK (IRTA) | South Africa (Zimbabwe)<br>South Africa | 2x<br>2x | ANG<br>ANG' |
| *C. dipsaceus* Ehrenb. ex Spach* | PI236468 | NCRPIS (IRTA) | East Africa (Ethiopia) | 2x | DIP |
| *C. prophetarum* L. | PI193967 | NCRPIS (IRTA) | East Africa (Ethiopia) | 4x | PRO |
| *C. sagittatus* Wawra & Peyr. | PI282441 | NCRPIS (IRTA) | South Africa (Namibia) | 2x | SAG |
| *C. zeyheri* Sond.* | PI282450 | NCRPIS (IRTA) | South Africa (Transvaal) | 2x | ZEY |
| *C. pustulatus* Naudin ex Hook.f.* | CUC44/1989[c] | IPK (IRTA) | East Africa | 2x | PUS |
| *C. globosus* C.Jeffrey | CUC76/1995 | IPK (IRTA) | South Africa (South Africa) | 2x | GLO |
| *C. ficifolius* A.Rich.* | BGV012786 /CEBAS-048 | COMAV | North-East Africa | 4x | FIC |
| *C. myriocarpus* Naudin | BGV008535/CEBAS-112 | COMAV | South Africa | 2x | MYR |
| *C. metuliferus* E.Mey. ex Naudin | BGV011135/CUC16/1982 | COMAV | South West Africa | 2x | MET |
| *C. melo* L.* | Ames24297/TRI 5-4 | NCRPIS (IRTA) | Pakistan | 2x | MEL |
| *C. maderaspatanus* Siddarthan s.n. | 322854 [*Mukia maderaspatana* (L.) M.Roem.] | Kew Garden | Mali | 2x | MAD |
| *C. zeyheri* × *C. anguria* | PI282450 × PI282442 | This work | ---- | 2x | Z×A |

| | | | | | |
|---|---|---|---|---|---|
| *C. dipsaceus* × *C. zeyheri* | PI236468 × PI282450 | This work | ---- | 2x | D×Z |
| *C. pustulatus* × *C. dipsaceus* | CUC44 × PI236468 | This work | ---- | 2x | P×D |
| *C. ficifolius* × *C. anguria* | BGV012786× PI282442 | This work | ---- | 3x | F×A |
| *C. ficifolius* × *C. dipsaceus* | BGV012786 × PI236468 | This work | ---- | 3x | F×D |
| *C. ficifolius* × *C. pustulatus* | BGV012786 × CUC44 | This work | ---- | 3x | F×P |
| *C. ficifolius* × *C. zeyheri* | BGV012786 × PI282450 | This work | ---- | 3x | F×Z |
| *C. dipsaceus* × *C. anguria* | PI236468 × CUC27 | This work | ---- | 2x | D×A’ |
| *C. anguria* × *C. anguria* | PI282442 × CUC27 / CUC27 × PI282442 | This work | ---- | 2x | A×A’/A’×A |

[a] NCPRIS: North Central Region Plant Introduction Station (USA); IRTA: Institut de Recerca i Tecnologia Agroalimentàries (Spain); IPK: Institut für Pflanzengenetik und Kulturpflanzenforschung (Germany); COMAV: Instituto de Conservación y Mejora de la Agrodiversidad Valenciana (Spain); Kew Garden: Royal Botanic Gardens, Kew (UK).
[b] Abbr.: Abbreviation.
[c] This accession is named as *C. figarei* by IPK germplasm bank but this is not an accepted name by [46]. We used instead *C. pustulatus* Naudin ex Hook.f. since phenotypic diagnostic traits correspond very well with this species.
* Accessions used to phenotype interspecific reproductive barriers.

**Supplementary Table S2**. Results of tests performed to determine statistical differences among means or mean ranks. Levene’s test for homogeneity of variance, Shapiro-Wilk normality test, and ANOVA or Kruskal Wallis chi squared test in case normality could not be assumed, for each pre-mating variable.

| | Levene’s test | | | Shapiro-Wilk test | | ANOVA | | | | | | | | Kruskal – Wallis test | | |
|---|---|---|---|---|---|---|---|---|---|---|---|---|---|---|---|---|
| | df | Fvalue | Pr(>F) | W | p-value | df | SS | MS | Fvalue | Res.df | Res. SS | Res. MS | Pr(>F) | $\chi^2$ | df | p-value |
| Pollen feret | 5 | 4.725 | <0.001 | 0.699 | <0.001 | - | - | - | - | - | - | - | - | - | - | - |
| Stigma length | 5 | 1.472 | 0.214 | 0.986 | 0.702 | 5 | 27.16 | 5.43 | 44.96 | 54 | 6.524 | 0.12 | <0.001 | - | - | - |
| Style length | 5 | 1.248 | 0.300 | 0.959 | 0.040 | - | - | - | - | - | - | - | - | 28.04 | 5 | <0.001 |
| Ovary length | 5 | 2.376 | 0.051 | 0.973 | 0.211 | 5 | 839.1 | 167.8 | 75.79 | 54 | 119.6 | 2.21 | <0.001 | - | - | - |

**Supplementary Table S2a**.Bonferroni-corrected pair-wise Mann-Whitney-Wilcoxon tests for non-homoscedastic and non-normal pollen feret data among *Cucumis* species.

| | *C. anguria* | *C. dipsaceus* | *C. ficifolius* | *C. melo* | *C. pustulatus* | *C. zeyheri* |
|---|---|---|---|---|---|---|
| *C. dipsaceus* | <0.001 | | | | | |
| *C. ficifolius* | <0.001 | <0.001 | | | | |
| *C. melo* | <0.001 | <0.001 | <0.001 | | | |
| *C. pustulatus* | <0.001 | <0.001 | <0.001 | <0.001 | | |
| *C. zeyheri* | <0.001 | 0.014 | <0.001 | <0.001 | <0.001 | |

**Supplementary Table S3**. Diagnostic traits measurement in leaves, male/female flowers and fruits of seed parent species and interspecific hybrids. Average ± standard deviation estimated from at least five measurements is shown for each trait.

| | Leaf | | | |
|---|---|---|---|---|
| **Taxon** | Pedicel (L) | Leaf (L) | Leaf (W) | Leaf (L/W) |
| C. anguria | 10.3 ± 1.3 | 15.7 ± 2.9 | 16.5 ± 1.1 | 0.9 ± 0.1 |
| C. pustulatus | 4.8 ± 0.6 | 10.3 ± 1.0 | 10.0 ± 0.3 | 1.0 ± 0.1 |
| C. ficifolius | 6.7 ± 1.0 | 8.5 ± 1.9 | 10.0 ± 1.0 | 0.8 ± 0.1 |
| C. dipsaceus | 10.0 ± 1.5 | 8.9 ± 0.9 | 11.6 ± 1.4 | 0.8 ± 0.04 |
| C. zeyheri | 4.1 ± 0.7 | 9.8 ± 0.2 | 8.8 ± 0.5 | 1.1 ± 0.03 |
| C. melo | 8.5 ± 1.3 | 9.9 ± 1.0 | 11.7 ± 1.3 | 0.8 ± 0.05 |
| ZxA | 7.6 ± 0.6 | 15.6 ± 2.6 | 16.7 ± 1.2 | 0.9 ± 0.1 |
| DxZ | 6.8 ± 0.8 | 9.6 ± 0.3 | 11.1 ± 0.4 | 0.9 ± 0.02 |
| PxD | 8.9 ± 1.0 | 9.6 ± 0.2 | 9.6 ± 0.3 | 1.0 ± 0.02 |
| FxA | 6.6 ± 0.9 | 12.5 ± 1.7 | 13.6 ± 1.0 | 0.9 ± 0.1 |
| FxD | 7.2 ± 1.1 | 12.5 ± 1.1 | 12.9 ± 0.8 | 1.0 ± 0.1 |
| FxP | 4.5 ± 0.5 | 9.8 ± 1.3 | 11.3 ± 0.6 | 0.9 ± 0.1 |
| FxZ | 4.2 ± 0.7 | 10.2 ± 0.8 | 10.8 ± 0.7 | 0.9 ± 0.03 |

| | Female flower | | | | | | | | |
|---|---|---|---|---|---|---|---|---|---|
| **Taxon** | Pedicel (L) | Ovary (L) | Ovary (W) | Ovary (L/W) | Sepal (L) | Petal (L) | Petal (W) | Petal (L/W) | Aculei hyaline/opaque (L)* |
| C. anguria | 3.8 ± 1.5 | 0.9 ± 0.1 | 0.6 ± 0.1 | 1.5 ± 0.2 | 0.4 ± 0.1 | 0.9 ± 0.1 | 0.7 ± 0.1 | 1.3 ± 0.3 | 0.5 ± 0.1 |
| C. pustulatus | 2.4 ± 0.1 | 1.3 ± 0.1 | 0.7 ± 0.1 | 1.7 ± 0.02 | 0.3 ± 0.03 | 1.0 ± 0.1 | 0.9 ± 0.06 | 1.1 ± 0.1 | 0.3 ± 0.07 |
| C. ficifolius | 1.4 ± 0.5 | 1.2 ± 0.1 | 0.6 ± 0.1 | 2.0 ± 0.2 | 0.3 ± 0.1 | 0.8 ± 0.1 | 0.7 ± 0.1 | 1.1 ± 0.1 | 1.6 ± 0.2 |
| C. dipsaceus | 2.0 ± 0.3 | 1.2 ± 0.1 | 0.7 ± 0.1 | 1.8 ± 0.1 | 0.6 ± 0.1 | 1.3 ± 0.2 | 1.0 ± 0.1 | 1.2 ± 0.1 | 1.7 ± 0.2 |
| C. zeyheri | 2.8 ± 0.7 | 1.3 ± 0.1 | 0.5 ± 0.1 | 2.5 ± 0.2 | 0.2 ± 0.04 | 1.0 ± 0.1 | 0.9 ± 0.1 | 1.2 ± 0.1 | 0.8 ± 0.2 |
| C. melo | 1.4 ± 0.2 | 0.8 ± 0.1 | 0.5 ± 0.04 | 1.6 ± 0.1 | 0.4 ± 0.1 | 0.8 ± 0.2 | 0.8 ± 0.1 | 1.0 ± 0.1 | --- |
| ZxA | 5.2 ± 1.1 | 1.1 ± 0.1 | 0.6 ± 0.1 | 1.8 ± 0.2 | 0.3 ± 0.1 | 1.0 ± 0.2 | 0.9 ± 0.1 | 1.1 ± 0.2 | 0.6 ± 0.05 |
| DxZ | 3.6 ± 1.5 | 1.7 ± 0.2 | 0.9 ± 0.1 | 1.9 ± 0.3 | 0.3 ± 0.1 | 1.3 ± 0.1 | 1.1 ± 0.2 | 1.3 ± 0.2 | 0.9 ± 0.1 |
| PxD | 3.7 ± 0.6 | 1.7 ± 0.2 | 1.0 ± 0.1 | 1.6 ± 0.1 | 0.6 ± 0.03 | 1.6 ± 0.1 | 1.3 ± 0.1 | 1.2 ± 0.1 | 1.8 ± 0.4 |
| FxA | 2.5 ± 0.5 | 1.1 ± 0.1 | 0.5 ± 0.1 | 2.1 ± 0.2 | 0.4 ± 0.04 | 1.2 ± 0.1 | 1.0 ± 0.1 | 1.2 ± 0.2 | 1.3 ± 0.2 |
| FxD | 2.0 ± 0.3 | 1.5 ± 0.1 | 0.8 ± 0.1 | 2.0 ± 0.2 | 0.5 ± 0.1 | 1.4 ± 0.2 | 1.1 ± 0.4 | 1.3 ± 0.5 | 1.1 ± 0.2 |
| FxP | 2.7 ± 1.0 | 1.4 ± 0.1 | 0.8 ± 0.1 | 1.9 ± 0.2 | 0.4 ± 0.1 | 1.2 ± 0.2 | 0.8 ± 0.2 | 1.6 ± 0.6 | 1.1 ± 0.1 |
| FxZ | Female sterile (did not produce female flowers) | | | | | | | | |

| | Male flower | | | | | | | |
|---|---|---|---|---|---|---|---|---|
| **Taxon** | Pedicel (L) | Calyx (L) | Calyx (W) | Calyx (L/W) | Sepal (L) | Petal (L) | Petal (W) | Petal (L/W) |
| C. anguria | 2.0 ± 0.6 | 0.5 ± 0.04 | 0.3 ± 0.1 | 1.4 ± 0.2 | 0.2 ± 0.1 | 0.9 ± 0.2 | 0.7 ± 0.1 | 1.3 ± 0.1 |
| C. pustulatus | 2.2 ± 0.6 | 0.5 ± 0.0 | 0.4 ± 0.02 | 1.2 ± 0.1 | 0.2 ± 0.04 | 1.0 ± 0.04 | 0.9 ± 0.0 | 1.1 ± 0.05 |
| C. ficifolius | 0.9 ± 0.1 | 0.6 ± 0.03 | 0.3 ± 0.04 | 1.7 ± 0.2 | 0.2 ± 0.02 | 0.7 ± 0.04 | 0.5 ± 0.04 | 1.2 ± 0.1 |
| C. dipsaceus | 2.4 ± 0.3 | 0.5 ± 0.04 | 0.3 ± 0.03 | 1.6 ± 0.1 | 0.4 ± 0.1 | 1.0 ± 0.1 | 0.9 ± 0.1 | 1.2 ± 0.2 |
| C. zeyheri | 2.5 ± 0.6 | 0.5 ± 0.03 | 0.4 ± 0.04 | 1.3 ± 0.2 | 0.2 ± 0.1 | 0.8 ± 0.1 | 0.7 ± 0.1 | 1.2 ± 0.2 |
| C. melo | 0.9 ± 0.1 | 0.4 ± 0.1 | 0.3 ± 0.02 | 1.4 ± 0.1 | 0.2 ± 0.03 | 0.8 ± 0.1 | 0.8 ± 0.1 | 1.0 ± 0.2 |
| ZxA | 1.7 ± 0.3 | 0.5 ± 0.04 | 0.4 ± 0.0 | 1.4 ± 0.2 | 0.3 ± 0.02 | 0.7 ± 0.1 | 0.7 ± 0.1 | 1.2 ± 0.2 |
| DxZ | Male sterile (did not produce male flowers) | | | | | | | |
| PxD | 2.1 ± 1.0 | 0.7 ± 0.05 | 0.4 ± 0.04 | 1.8 ± 0.1 | 0.3 ± 0.06 | 1.1 ± 0.2 | 1.0 ± 0.2 | 1.1 ± 0.1 |
| FxA | Male sterile (did not produce male flowers) | | | | | | | |
| FxD | Male sterile (did not produce male flowers) | | | | | | | |
| FxP | 0.8 ± 0.1 | 0.5 ± 0.04 | 0.3 ± 0.02 | 1.7 ± 0.2 | 0.1 ± 0.04 | 0.5 ± 0.1 | 0.5 ± 0.1 | 1.0 ± 0.2 |
| FxZ | Male sterile (did not produce male flowers) | | | | | | | |

| | Fruit** | | | | | | |
|---|---|---|---|---|---|---|---|
| **Taxon** | Peduncle (L) | Peduncle d/p (W)*** | Aculei (L) | Fruit (L) | Fruit (W) | Fruit (L/W) | Seed number/ fruit |
| C. anguria | 7.8 ± 1.9 | 1.9 ± 0.5 | 0.9 ± 0.2 | 4.2 ± 0.3 | 3.8 ± 0.2 | 1.1 ± 0.03 | 147.2 ± 34.3 |
| C. pustulatus | 10.1 ± 2.6 | 3.2 ± 0.6 | 0.6 ± 0.3 | 7.4 ± 0.8 | 5.4 ± 0.5 | 1.4 ± 0.1 | 652.8 ± 294.1 |
| C. ficifolius | 2.8 ± 0.4 | 1.3 ± 0.2 | 0.06 ± 0.03 | 4.7 ± 0.6 | 3.4 ± 0.4 | 1.4 ± 0.1 | 175.3 ± 61.7 |
| C. dipsaceus | 2.7 ± 0.6 | 1.4 ± 0.3 | 0.5 ± 0.1 | 5.9 ± 0.7 | 3.3 ± 0.5 | 1.8 ± 0.2 | 523.0 ± 202.3 |
| C. zeyheri | 5.6 ± 1.3 | 1.4 ± 0.2 | 0.5 ± 0.1 | 7.6 ± 0.3 | 4.3 ± 0.1 | 1.8 ± 0.1 | 167.7 ± 22.1 |
| C. melo | 2.6 ± 0.5 | 1.8 ± 0.5 | --- | 3.9 ± 0.6 | 3.4 ± 0.2 | 1.1 ± 0.1 | 158.0 ± 31.1 |
| ZxA | 6.5 ± 1.2 | 1.8 ± 0.3 | 0.4 ± 0.1 | 4.2 ± 0.3 | 2.8 ± 0.3 | 1.5 ± 0.1 | 77.5 ± 9.3 |
| DxZ | 3.2 ± 1.2 | 0.9 ± 0.4 | 0.6 ± 0.1 | 6.3 ± 0.3 | 3.0 ± 0.4 | 2.2 ± 0.4 | 123.0 ± 7.1 |
| PxD | 9.1 ± 1.8 | 2.8 ± 0.6 | 0.8 ± 0.03 | 8.0 ± 0.8 | 5.7 ± 0.6 | 1.4 ± 0.1 | 452.7 ± 243.9 |
| FxA | 3.0 ± 1.0 | 1.3 ± 0.2 | 0.4 ± 0.1 | 3.5 ± 0.1 | 2.3 ± 0.2 | 1.5 ± 0.1 | 0 |
| FxD | 1.9 ± 0.2 | 1.5 ± 0.3 | 0.2 ± 0.02 | 4.3 ± 0.3 | 2.3 ± 0.4 | 1.9 ± 0.3 | 22.0 ± 13.2 |
| FxP | Only one small fruit without seeds was obtained by backcrossing | | | | | | |
| FxZ | Female sterile (did not produce female flowers) | | | | | | |

L (length in cm)
W (width in cm)
* Relative length of the apical hyaline bristle to the opaque basal portion of the aculei
** Fruits were obtained by self-fertilization in seed parent species and by backcrossing in male-sterile hybrids
***Peduncle (distal/proximal thickness ratio)

**Supplementary Table S4**. Crossability between *C. anguria* accessions PI282442 (ANG) and CUC27/1983 (ANG').

| Cross | Fuit set | Fruit Weight | Seed number | Germination | F1 hybrid | F1 hybrid self-pollination |
|---|---|---|---|---|---|---|
| ANG self-pollination | 80% | 10,4 ± 0,7 | 164,7 ± 10,8 | 75% | | |
| ANG X ANG' | 67% | 11,5 ± 0,4 | 170,4 ± 29,5 | 80% | male fertile | verified |
| ANG' self-pollination | 50% | 5,9 ± 2,4 | 38,3 ± 15,2 | 63% | | |
| ANG' X ANG | 80% | 8,5 ± 2,70 | 59,8 ± 14,6 | 58% | male fertile | verified |

**Supplementary Table S5**. Percentage of heterozygous sites detected in the *Cucumis* taxa analyzed. Proportion of heterozygous sites was estimated from the set of 10967 SNPs used to infer genetic distances among *Cucumis* species.

| Taxa | Number of sites | Number heterozygous | Proportion heterozygous | % |
|---|---|---|---|---|
| MEL | 10967 | 3 | 0,0003 | 0,03 |
| LON | 10967 | 30 | 0,0027 | 0,27 |
| MAD | 10967 | 37 | 0,0034 | 0,34 |
| SAG | 10967 | 40 | 0,0036 | 0,36 |
| MET | 10967 | 28 | 0,0026 | 0,26 |
| GLO | 10967 | 43 | 0,0039 | 0,39 |
| ANG | 10967 | 29 | 0,0026 | 0,26 |
| PUS | 10967 | 31 | 0,0028 | 0,28 |
| DIP | 10967 | 35 | 0,0032 | 0,32 |
| ZEY | 10967 | 45 | 0,0041 | 0,41 |
| MYR | 10967 | 29 | 0,0026 | 0,26 |
| FIC | 10967 | 1417 | 0,1292 | 12,92 |
| PRO | 10967 | 1407 | 0,1283 | 12,83 |
| DXZ | 10967 | 1107 | 0,1009 | 10,09 |
| ZXA | 10967 | 1124 | 0,1025 | 10,25 |
| PXD | 10967 | 1478 | 0,1348 | 13,48 |
| LXA | 10967 | 174 | 0,0159 | 1,59 |
| DXL | 10967 | 1046 | 0,0954 | 9,54 |
| FXD | 10967 | 1916 | 0,1747 | 17,47 |
| FXA | 10967 | 1998 | 0,1822 | 18,22 |
| FXZ | 10967 | 1980 | 0,1805 | 18,05 |
| FXP | 10967 | 2139 | 0,1950 | 19,50 |
| A'XA | 10967 | 174 | 0,0159 | 1,59 |

**Supplementary Table S6**. Pre-zygotic (pollen-pistil compatibility) RI indices for all species pairs, where RI = 1- (average success of interspecific crosses/average success of self-pollinations).

**Pollen-pistil compatibility**

| | | **Pollen parent** | | | | | |
|---|---|---|---|---|---|---|---|
| | ø | **ANG** | **ZEY** | **DIP** | **PUS** | **FIC** | **MELO** |
| **Seed Parent** | **ANG** | 0.00 | 0.35 | 0.80 | 0.88 | 0.75 | 0.75 |
| | **ZEY** | 0.00 | 0.00 | 0.48 | 1.00 | 0.75 | 0.63 |
| | **DIP** | 0.23 | 0.31 | 0.00 | 1.00 | 0.69 | 0.85 |
| | **PUS** | 0.00 | 0.19 | 0.13 | 0.00 | 0.28 | 0.31 |
| | **FIC** | 0.04 | 0.00 | 0.00 | 0.20 | 0.00 | 0.41 |
| | **MEL** | 0.91 | 0.89 | 0.89 | 0.69 | 1.00 | 0.00 |

**Supplementary Table S7**. Post-zygotic (fruit set) RI indices for all species pairs, where RI = 1 - (average success of interspecific crosses/average success of self-pollinations).

**Fruit set**

| | | **Pollen parent** | | | | | |
|---|---|---|---|---|---|---|---|
| | ø | **ANG** | **ZEY** | **DIP** | **PUS** | **FIC** | **MEL** |
| **Seed Parent** | **ANG** | **0.00** | **0.56** | **0.52** | **1.00** | **1.00** | **1.00** |
| | **ZEY** | 0.18 | 0.00 | 1.00 | 1.00 | 1.00 | 1.00 |
| | **DIP** | 0.95 | 0.56 | 0.00 | 1.00 | 1.00 | 1.00 |
| | **PUS** | 1.00 | 1.00 | 0.14 | 0.00 | 1.00 | 1.00 |
| | **FIC** | 0.00 | 0.00 | 0.00 | 0.59 | 0.00 | 1.00 |
| | **MEL** | 1.00 | 1.00 | 1.00 | 1.00 | 1.00 | 0.00 |

**Supplementary Table S8**. Post-zygotic (fruit weight) RI indices for all species pairs, where RI = 1 - (average success of interspecific crosses/average success of self-pollinations).

**Fruit weight**

| | | **Pollen parent** | | | | | |
|---|---|---|---|---|---|---|---|
| | ø | **ANG** | **ZEY** | **DIP** | **PUS** | **FIC** | **MEL** |
| **Seed Parent** | **ANG** | 0.00 | 0.63 | 0.55 | 1.00 | 1.00 | 1.00 |
| | **ZEY** | 0.00 | 0.00 | 1.00 | 1.00 | 1.00 | 1.00 |
| | **DIP** | 0,48 | 0.40 | 0.00 | 1.00 | 1.00 | 1.00 |
| | **PUS** | 1.00 | 1.00 | 0.00 | 0.00 | 1.00 | 1.00 |
| | **FIC** | 0.03 | 0.20 | 0.19 | 0.55 | 0.00 | 1.00 |
| | **MEL** | 1.00 | 1.00 | 1.00 | 1.00 | 1.00 | 0.00 |

**Supplementary Table S9**. Post-zygotic (seed set) RI indices for all species pairs, where RI = 1 - (average success of interspecific crosses/average success of self-pollinations).

**Seed set**

| | | **Pollen parent** | | | | | |
|---|---|---|---|---|---|---|---|
| | ø | **ANG** | **ZEY** | **DIP** | **PUS** | **FIC** | **MEL** |
| **Seed Parent** | **ANG** | 0.00 | 0.87 | 0.79 | 1.00 | 1.00 | 1.00 |
| | **ZEY** | 0.07 | 0.00 | 1.00 | 1.00 | 1.00 | 1.00 |
| | **DIP** | 0.91 | 0.73 | 0.00 | 1.00 | 1.00 | 1.00 |
| | **PUS** | 1.00 | 1.00 | 0.11 | 0.00 | 1.00 | 1.00 |
| | **FIC** | 0.00 | 0.00 | 0.00 | 0.46 | 0.00 | 1.00 |
| | **MEL** | 1.00 | 1.00 | 1.00 | 1.00 | 1.00 | 0.00 |
